# Artificial intelligence-based multimodal prediction for nuclear grading status and prognosis of clear cell renal cell carcinoma: a multicenter cohort study

**DOI:** 10.1097/JS9.0000000000002368

**Published:** 2025-03-28

**Authors:** Qingyuan Zheng, Haonan Mei, Xiaodong Weng, Rui Yang, Panpan Jiao, Xinmiao Ni, Xiangxiang Yang, Jiejun Wu, Junjie Fan, Jingping Yuan, Xiuheng Liu, Zhiyuan Chen

**Affiliations:** a Department of Urology, Renmin Hospital of Wuhan University, Wuhan, Hubei, China; b Institute of Urologic Disease, Renmin Hospital of Wuhan University, Wuhan, Hubei, China; c University of Chinese Academy of Sciences, Beijing, China; d Trusted Computing and Information Assurance Laboratory, Institute of Software, Chinese Academy of Sciences, Beijing, China; e Department of Pathology, Renmin Hospital of Wuhan University, Wuhan, Hubei, China

**Keywords:** deep learning, multimodal, pathology, radiology, renal cell carcinoma

## Abstract

**Background:**

The assessment of the International Society of Urological Pathology (ISUP) nuclear grade is crucial for the management and treatment of clear cell renal cell carcinoma (ccRCC). This study aimed to explore the value of using integrated multimodal information for ISUP grading and prognostic stratification in ccRCC patients, to guide postoperative adjuvant therapy.

**Methods:**

This retrospective study analyzed a total of 729 patients from three cohorts, utilizing whole slide i-mages and computed tomography (CT) images. Artificial intelligence algorithms were used to extract morphological and textural features from whole slide images and CT images separately, creating single-modality predictive models for ISUP grading. By combining the CT and pathology single-modality predictive features, a multimodal predictive signature (MPS) was developed. The prognostic performance of the MPS model was further validated in two independent cohorts.

**Results:**

The single-modality predictive models for CT and pathology performed well in predicting ISUP grade for ccRCC. The MPS model achieved higher area under the curve values of 0.95, 0.93, and 0.95 across three independent patient cohorts. Additionally, the MPS model was able to distinguish patients with poorer overall survival. In the external validation cohort, uni- and multivariate analyses showed hazard ratios of 2.542 (95% confidence interval [CI]: 1.363–4.741, *P* < 0.0001) and 1.723 (95% CI: 0.888–3.357, *P* = 0.003), respectively. The C-index values for the two cohorts were 0.75 and 0.71. Furthermore, the MPS outperformed single-modality models, providing a complementary tool for current risk stratification in ccRCC adjuvant therapy.

**Conclusion:**

Our novel MPS model demonstrated high accuracy in ISUP grading for ccRCC patients. With further validation across multiple centers, the MPS model could be used for precise detection of nuclear grading in ccRCC, serving as an effective tool for assisting clinical decision-making.

**HIGHLIGHTS**

- We proposed a multimodal prediction model based on computed tomography images and pathology images to accurately diagnose the International Society of Urological Pathology (ISUP) grading status of clear cell renal cell carcinoma.
- The diagnostic accuracy and sensitivity of the multimodal model were superior to those of unimodal models.
- The ISUP grading diagnosed by the multimodal model could predict the overall survival status of clear cell renal cell carcinoma patients, aiding in risk stratification and personalized treatment decision-making.

## Introduction

Clear cell renal cell carcinoma (ccRCC) is the most common pathological subtype of renal cell carcinoma, accounting for approximately 90% of all cases^[^1^]^. In recent years, the mortality rate of ccRCC has been increasing, highlighting its importance in clinical diagnosis, treatment, and prognosis^[^2,3^]^. The prognosis of ccRCC patients is closely related to the nuclear grade of the tumor^[^4,5^]^. The World Health Organization/International Society of Urological Pathology grading system, established in 2016, is widely used to classify ccRCC into grades I–IV^[^6^]^. Additionally, a simplified two-tier grading system, which categorizes ISUP I-II as low-grade and ISUP III-IV as high-grade, is more clinically practical. High-grade ccRCC is more aggressive, prone to metastasis, and has a poorer prognosis^[^7^]^. Therefore, determining tumor grade can help guide appropriate personalized treatment decisions and clinical strategies.

Renal multiphase contrast-enhanced CT scan is a commonly used non-invasive imaging technique for tumor staging and assessing the aggressiveness of ccRCC^[^8,9^]^. However, its ability to differentiate between high-grade and low-grade ccRCC is limited. Histopathology is the gold standard for diagnosis, but there is interobserver variability among pathologists in assessing renal cancer nuclear grade, with only moderate consistency scores^[^10^]^. Therefore, the development of new tools to assist evaluation could be valuable in improving grading accuracy and follow-up management. Artificial intelligence (AI)-based radiomics and pathomics are emerging technologies that provide solutions by transforming medical images into quantitative features that can be analyzed using advanced modeling algorithms^[^11–13^]^. Previous studies have demonstrated that single-modality models based on radiology or pathology have shown decent prospects for use in ccRCC, particularly for diagnosing Fuhrman grading, predicting prognosis, and assessing treatment response^[^14–16^]^. While single-modality features can improve the diagnostic performance of ISUP grading for ccRCC to some extent, integrating multimodal information may further enhance the predictive accuracy of cancer grading^[^17^]^.

In this study, we developed and validated a multimodal AI diagnostic system for ISUP grading in ccRCC patients based on features from pathomics and radiomics. The AI model demonstrated high performance in diagnosing nuclear grade across multiple cohorts, showing great potential for clinical application. Additionally, the ISUP grade diagnosed by the AI model had significant prognostic value for ccRCC patients.

## Methods

### Patient cohorts and data resource

**Figure 1.**
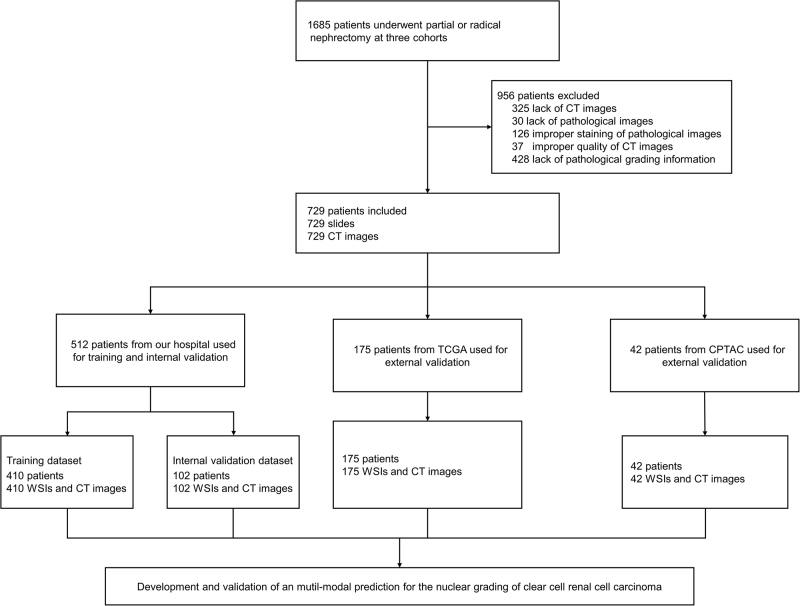
Flowchart of development of multimodal predictive model. CPTAC, Clinical Proteomic Tumor Analysis Consortium; CT, computed tomography; TCGA, The Cancer Genome Atlas; WSI, whole slide image.

This study retrospectively included three cohorts of ccRCC patients. The inclusion criteria were: (1) a confirmed pathological diagnosis of ccRCC; (2) no other types of malignancies; (3) availability of CT urography (CTU) images, whole slide images (WSIs), and clinicopathological information. In the General cohort, 512 ccRCC patients who underwent radical or partial nephrectomy at our hospital between June 2016 and June 2024 were included. Additionally, 175 ccRCC patients were recruited from The Cancer Genome Atlas (TCGA) cohort, with complete access to hematoxylin and eosin (H&E)-stained WSIs, CTU images, and overall survival (OS) information. Lastly, this study included another 42 ccRCC patients from the Clinical Proteomic Tumor Analysis Consortium (CPTAC cohort) who met the above inclusion criteria, providing H&E-stained images, CTU images, and survival information. OS was defined as the time from initial surgery to death from any cause or the last follow-up. Basic clinical information of the included patients is shown in Table 1. The patient inclusion process is shown in Figure 1. This study was conducted in accordance with the Helsinki Declaration and reported following the Standards for Reporting Diagnostic Accuracy^[^18^]^.

**Table 1:** Clinicopathologic baseline characteristics of included patients with ccRCC.

	General cohort	TCGA cohort	CPTAC cohort
Number of patients	512	175	42
Age (years)	58 (12, 87)	59 (26, 88)	65 (41, 90)
Gender			
Female	193 (37.7%)	64 (36.6%)	15 (35.7%)
Male	319 (62.3%)	111 (63.4%)	27 (64.3%)
Laterality			
Right	285 (55.7%)	88 (50.3%)	-
Left	227 (44.3%)	87 (49.7%)	-
Histological type			
ccRCC	512 (100%)	175 (100%)	42 (100%)
pT stage			
pT1	389 (76.0%)	92 (52.5%)	10 (23.8%)
pT2	41 (8.0%)	19 (10.9%)	6 (14.3%)
pT3	69 (13.5%)	61 (34.9%)	7 (16.7%)
pT4	13 (2.5%)	3 (1.7%)	1 (2.4%)
pTx	0	0	18 (42.8%)
pN stage			
pN0	492 (96.1%)	74 (42.3%)	2 (4.8%)
pN1	20 (3.9%)	3 (1.7%)	0
pNx	0	98 (56.0%)	40 (95.2%)
pM stage			
pM0	509 (99.4%)	144 (82.3%)	4 (9.5%)
pM1	3 (0.6%)	25 (14.3%)	0
pMx	0	6 (3.4%)	38 (90.5%)
pTNM stage			
Stage I	390 (76.2%)	89 (50.9%)	17 (40.5%)
Stage II	40 (7.8%)	15 (8.6%)	5 (11.9%)
Stage III	67 (13.1%)	44 (25.1%)	12 (28.6%)
Stage IV	15 (2.9%)	27 (15.4%)	4 (9.5%)
Missing	0	0	4 (9.5%)
ISUP grade			
I	133 (26.0%)	0	3 (7.1%)
II	293 (57.2%)	71 (40.6%)	24 (57.1%)
III	69 (13.5%)	77 (44.0%)	12 (28.6%)
IV	17 (3.3%)	27 (15.4%)	3 (7.2%)

ccRCC, clear cell renal cell carcinoma; CPTAC, Clinical Proteomic Tumor Analysis Consortium; ISUP, International Society of Urological Pathology; TCGA, The Cancer Genome Atlas.

### Radiomics-based CTU image analysis

Conventional CTU images include the non-enhanced phase, corticomedullary phase (CMP), nephrographic phase, and excretory phase. Previous study had identified CMP as the optimal phase for diagnosing ISUP nuclear grade, where the intensity difference between high-grade and low-grade tumors was most pronounced, and the radiomics model performance was more robust^[^19^]^. Considering data completeness across all cohorts, we uniformly used CMP images for analysis. Two senior experts, each with 12 years of experience, manually delineated the renal tumors on each CTU image as regions of interest (ROI) using ITK-SNAP (version 3.6.0). In cases of ambiguity, a third expert with 20 years of experience reviewed and made the final assessment.

After tumor segmentation, radiomic features were extracted from the ROI using the PyRadiomics package. A total of 128 features were calculated from ROI of each CTU image. The least absolute shrinkage and selection operator (LASSO) regression was then applied to select the optimal subset of features. Ultimately, LASSO regression identified eight radiomic features which were used to build the radiomics-based signature (RBS) model (Supplemental Digital Content Table S1, available at: http://links.lww.com/JS9/E38).

### Pathomics-based WSI image analysis

In this study, we developed and validated a pathomic-based signature (PBS) model for diagnosing the ISUP nuclear grade status of ccRCC patients. Using QuPath software (version 0.5), tumor regions in each WSI were manually outlined. The built-in segmentation function of QuPath was then used to divide the tumor regions into 512 × 512 patches, followed by color normalization of all patches using the Vahadane method^[^20^]^. We employed the Hover-Net model to locate nuclei within each patch and classify their cell types. Hover-Net is a deep convolutional neural network that simultaneously segments and classifies nuclei on WSIs^[^21^]^. The model was pre-trained on the public PanNuke dataset and can identify four types of nuclei: tumor cells, inflammatory cells, connective cells, and dead/necrotic nuclei. Examples of segmentation can be found in the Supplemental Digital Content Figure S1, available at: http://links.lww.com/JS9/E38. For each patch, Hover-Net output information about the centroids, contours, and types of the detected nuclei, which was then used for subsequent feature extraction.

By accurately identifying tumor cells using Hover-Net, we extracted two categories of features for individual nuclei: 14 morphological features and 9 textural features. (1) Morphological features were calculated to describe the shape and contour of the nuclei. By generating masks based on the nuclei contour coordinates, the following 14 morphological features were extracted. (2) Textural features were calculated to characterize the local pixel distribution patterns within the nuclear contours. By converting the color image to grayscale and extracting nuclear patches based on the bounding box, a gray-level co-occurrence matrix was calculated for each nucleus, from which five features were derived. Additionally, four statistical measures of pixel intensity within the nuclear contours were computed. Supplemental Digital Content Table S7, available at: http://links.lww.com/JS9/E38, provides detailed descriptions of all morphological and textural features. We performed LASSO regression to identify 16 pathological features which were used to build the classification model.

### Multimodal prediction of ISUP grading status

**Figure 2.**
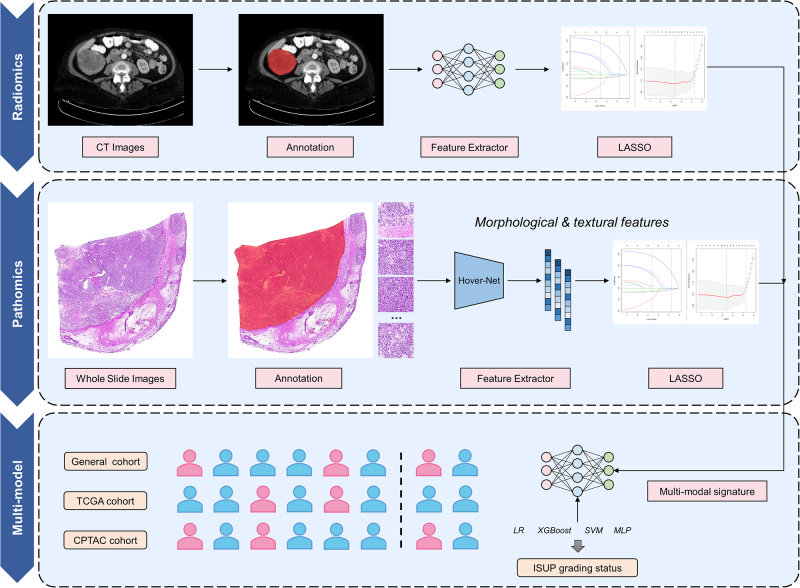
Study design and analysis process of this study. CPTAC, Clinical Proteomic Tumor Analysis Consortium; CT, computed tomography; ISUP, International Society of Urological Pathology; LASSO, least absolute shrinkage and selection operator; TCGA, The Cancer Genome Atlas.

By applying support vector machine (SVM), multilayer perceptron (MLP), logistic regression (LR), and XGBoost classifiers to integrate multimodal features, we developed a multi-predictive signature (MPS) model based on computed tomography (CT) and pathological features from the General cohort (Supplemental Digital Content Table S3, available at:http://links.lww.com/JS9/E38). The models were trained using five-fold cross-validation. We further validated the clinical value of the MPS system in two independent cohorts (TCGA and CPTAC cohorts). Additionally, the output probabilities from the best-performing MPS model were saved for subsequent survival analysis. To enhance model interpretability, we quantified the impact of each feature on the model predictions by analyzing Shapley Additive Explanations (SHAP) values. A detailed design of the study is illustrated in Figure 2.

### Statistical analysis

All statistical analyses were performed using R software (version 3.5.1) and IBM SPSS (version 26.0). The diagnostic performance of each model was assessed using receiver operating characteristic (ROC) curves and the area under the curve (AUC). Kaplan–Meier survival curves were used to calculate the survival functions of the two patient groups, and differences were compared using the log-rank test. The Harrell’s concordance index (C-index) was used to evaluate the ability of the MPS to predict OS. Univariate and multivariate Cox proportional hazards models were employed to assess the relationships between the MPS, clinical variables and OS. A two-tailed *P*-value of less than 0.05 was considered statistically significant.

## Results

### Performance of single-modality prediction models for ISUP grading

The radiomics-based RBS model demonstrated strong diagnostic performance, achieving an AUC of 0.93 (95% CI: 0.90–0.96) and an accuracy of 0.94 (95% CI: 0.90–0.98) in the training set. Validation results showed AUCs of 0.89 (95% CI: 0.82–0.94), 0.83 (95% CI: 0.79–0.88), and 0.88 (95% CI: 0.84–0.92) with corresponding accuracies of 0.86, 0.86, and 0.79 in the internal, TCGA, and CPTAC cohorts, respectively, confirming its reliability as a non-invasive tool for ISUP grading using preoperative CT images.

The pathomics-based PBS model, focusing on nuclear segmentation, achieved an AUC of 0.94 (95% CI: 0.90–0.99) and an accuracy of 0.96 (95% CI: 0.92–0.99) in the training set. In validation, it achieved AUCs of 0.90 (95% CI: 0.86–0.94), 0.88 (95% CI: 0.84–0.92), and 0.89 (95% CI: 0.85–0.92), with accuracies of 0.91, 0.90, and 0.88 across the internal, TCGA, and CPTAC cohorts, demonstrating robust performance for ISUP grading (Supplemental Digital Content Table S5, available at: http://links.lww.com/JS9/E38).

### Development and validation of the MPS model for ISUP grading in ccRCC patients

**Figure 3.**
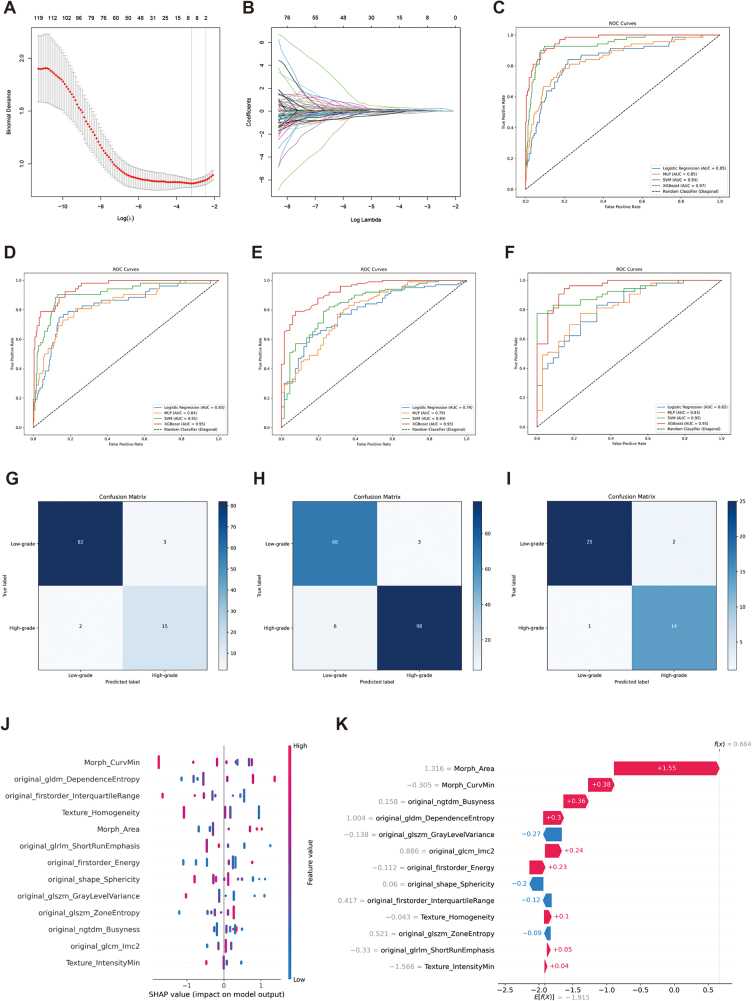
Development process and validation results of the MPS model. (A, B) LASSO dimensionality reduction result plots. (C–F) Receiver operating characteristic analysis of the MPS model in the training set, internal validation set, TCGA cohort, and CPTAC cohort. (G–I) Confusion matrix plots for the MPS model in the internal validation set, TCGA cohort, and CPTAC cohort. (J, K) SHAP analysis illustrating the contribution of each feature in the MPS model predictions. CPTAC, Clinical Proteomic Tumor Analysis Consortium; LASSO, least absolute shrinkage and selection operator; MPS, multimodal predictive signature; SHAP, Shapley additive explanations; TCGA, The Cancer Genome Atlas.

We developed the MPS model using four classifiers (SVM, LR, MLP, and XGBoost) based on radiological and pathological features from the training set (general cohort). The XGBoost model, which demonstrated the highest accuracy in predicting ISUP grade for ccRCC patients, was selected as the final MPS model (Fig. 3). In the training set, the MPS model achieved an AUC of 0.97 (95% CI: 0.94–0.99) and an accuracy of 0.98 (95% CI: 0.95–1.00). Validation in the internal set, TCGA, and CPTAC cohorts showed AUCs of 0.95 (95% CI: 0.92–0.97), 0.93 (95% CI: 0.89–0.96), and 0.95 (95% CI: 0.90–0.98), with accuracies of 0.95 (95% CI: 0.92–0.98), 0.95 (95% CI: 0.91–0.98), and 0.93 (95% CI: 0.90–0.96), respectively (Table 2). A comparative analysis across the entire dataset confirmed that the MPS model outperformed single-modality models (*P* < 0.001, DeLong test).

**Table 2:** The diagnostic performance of multimodel prediction in different cohorts.

	TP	FN	TN	FP	Accuracy (95% CI)	Sensitivity (95% CI)	Specificity (95% CI)	AUC (95% CI)
General training cohort	66	3	337	4	0.98 (0.95–1.00)	0.96 (0.92–0.99)	0.99 (0.96–1.00)	0.97 (0.94–0.99)
General validation cohort	15	2	82	3	0.95 (0.92–0.98)	0.88 (0.82–0.95)	0.96 (0.93–0.98)	0.95 (0.92–0.97)
TCGA validation cohort	98	6	68	3	0.95 (0.91–0.98)	0.94 (0.90–0.98)	0.96 (0.90–0.99)	0.93 (0.89–0.96)
CPTAC validation cohort	14	1	25	2	0.93 (0.90–0.96)	0.93 (0.89–0.97)	0.93 (0.90–0.97)	0.95 (0.90–0.98)

CPTAC, Clinical Proteomic Tumor Analysis Consortium; FN, false negative; FP, false positive; TCGA, The Cancer Genome Atlas; TN, true negative; TP, true positive.

### Interpretability of the MPS model

For the optimal XGBoost model, we visualized feature decisions using SHAP values, which assess the importance of individual features in model predictions. As shown in Figure 3, “Morph_Area” had the greatest impact on predictions, followed by “Morph_CurvMin,” “ngtdm_Busyness,” “gldm_DependenceEntropy” and others. Features like “glszm_GrayLevelVariance” and “shape_Sphericity” showed an inverse relationship, suggesting that higher gray-level variance and sphericity in CT images are associated with lower pathological grades, reflecting more regular morphology in lower-grade tumors. Conversely, “Morph_Area,” “Morph_CurvMin,” “ngtdm_Busyness,” and “gldm_DependenceEntropy” exhibited a direct relationship with higher grades, indicating that larger nuclear area, greater curvature, and increased gray-level variation in CT images are linked to higher pathological grades. These findings suggest that higher-grade tumors have more complex tissue structures and greater heterogeneity, which are associated with increased malignancy and aggressiveness.

### Prognostic value of MPS in ccRCC patients

**Figure 4.**
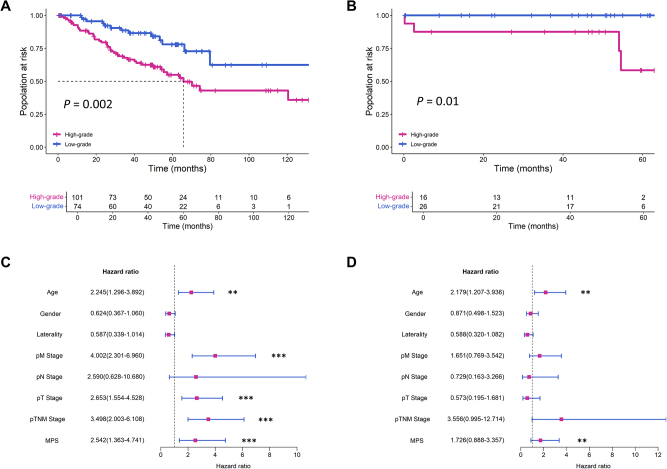
Development process and validation results of the MPS model. (A, B) Kaplan–Meier curve showing the survival stratification of RCC patients based on MPS-predicted grading status in the TCGA and CPTAC cohort, respectively. (C, D) Forest plot of univariate and multivariate Cox proportional hazards analysis in the TCGA cohort, with age, gender, laterality, pT, pN, Pm, and pTNM stage as covariates, respectively. CPTAC, Clinical Proteomic Tumor Analysis Consortium; MPS, multimodal predictive signature; TCGA, The Cancer Genome Atlas.

The TCGA and CPTAC cohorts included 175 and 42 ccRCC patients, respectively, with median survival times of 44.6 months (±30.7) and 41 months (±20.6). Given the association between ISUP grading and survival outcomes, we hypothesized that the MPS model’s predictions could correlate with patient prognosis. Kaplan–Meier survival analysis, using the log-rank test, demonstrated that the MPS model effectively stratified patients based on OS in both cohorts (*P* = 0.002 and 0.01, respectively; Fig. 4). The C-index for MPS in the TCGA and CPTAC cohorts was 0.75 and 0.71, respectively. Cox proportional hazard analysis, adjusting for age, gender, laterality, T stage, N stage, M stage, and TNM stage, revealed that in the TCGA cohort, the univariate hazard ratio for MPS was 2.542 (95% CI: 1.363–4.741; *P* < 0.001), and the multivariate hazard ratio was 1.723 (95% CI: 0.888–3.357; *P* = 0.003; Fig. 4). Subgroup analysis confirmed MPS as a strong independent prognostic factor across variables such as age, gender, and tumor stage (Supplemental Digital Content Figure S4, available at: http://links.lww.com/JS9/E38). These findings demonstrate the prognostic value of the ISUP status predicted by the MPS model for ccRCC patients.

## Discussion

Accurate histological grading is critical for monitoring ccRCC progression and devising personalized follow-up treatment strategies^[^22^]^. In this study, we developed an MPS model that combines radiological and pathological features to predict the ISUP nuclear grade of ccRCC. The model performed well in two external validation sets, achieving AUCs of 0.93 and 0.95, and demonstrated superior diagnostic performance compared to classical single-modality models. Moreover, the nuclear grade predictions from the MPS model showed significant prognostic value. To our knowledge, this is the first study using a multimodal approach for automated ccRCC grading assessment.

Previous research has highlighted the potential challenges of digital grading for RCC. For instance, Browning *et al* reported interobserver variability in nuclear grade assessments by different pathologists, with only moderate agreement scores^[^10^]^. Enhanced renal CT scans are essential for the early radiological diagnosis of ccRCC, and studies have indicated that the degree of dynamic enhancement in the images is a crucial indicator for diagnosing renal malignancies^[^23,24^]^. However, it can be challenging for clinicians to visually discern subtle differences in intensity uniformity across different enhancement phases, which may lower diagnostic efficiency. With the rapid development of AI, grading models based on medical image segmentation and feature extraction are becoming more practical. For example, deep learning models can automatically identify and quantify radiological and pathological features of tumors, providing objective support for grading and prognosis^[^14,19,24–26^]^. Analyses using these features can effectively distinguish different grades of ccRCC, thereby supporting clinical decision-making.

Over the past 5 years, research on ccRCC diagnostic assistance has made significant progress. When utilizing medical AI analysis, four primary types of images are commonly involved: ultrasound, CT, MRI, and pathological images. In 2024, Luo *et al* employed a unimodal machine learning model based on ultrasound images, achieving an external test AUC of 0.8111 on data from 122 patients^[^27^]^. For CT images, five studies have reported the feasibility of using AI models to predict ISUP grading. The earliest study, conducted by Shu *et al* in 2019, analyzed data from 271 patients and reported an external test AUC of 0.856^[^28^]^. Another study by Sun *et al*, involving 227 patients, reported an AUC of 0.88^[^29^]^. As of 2024, only three related studies have been published, with reported AUC values ranging between 0.8 and 0.9 despite limited data availability. Regarding MRI images, a single study in 2020 confirmed the feasibility of ISUP grade identification. Li *et al* included data from 379 patients, achieving an AUC of 0.842^[^30^]^. For pathological images, fewer studies exist. Yang *et al* analyzed data from 90 patients and demonstrated the feasibility of diagnosing ISUP grade from pathological images^[^31^]^.

Unlike previous studies, our research integrated multiple factors, including pathological and radiological features, to develop a more accurate diagnostic model for ccRCC patients. The microscopic phenotypes, such as the morphology and texture of renal carcinoma cells, can be reflected by WSIs, while the macroscopic phenotypes, such as tumor necrosis, morphological texture changes, and adjacent area involvement, can be reflected by CT images. Compared to classical models based on radiomics or pathomics alone, this novel multimodal approach demonstrated higher diagnostic accuracy and generalization performance. This suggests that integrating multiple factors into a single predictive model warrants further investigation.

Grading of ccRCC is closely linked to tumor biology and patient prognosis^[^32^]^. ISUP grading is incorporated into patient risk stratification system, such as the Leibovich score, which predicts the likelihood of local RCC progressing to metastatic disease and influences patient prognosis^[^33^]^. Our analysis in the TCGA and CPTAC cohorts confirmed the capability of MPS in predicting ISUP grade and its prognostic value. AI-based predictions identified MPS as a significant prognostic factor for ccRCC patients, with univariate and multivariate Cox analyses demonstrating that it is independent of existing clinical and pathological stage parameters.

Thus, our MPS model could be seamlessly integrated into routine clinical workflows through the following applications. First, automating tumor grading and prognostic analysis during intraoperative assessments, offering real-time decision support to surgical teams. Second, serving as a second-opinion tool for clinicians evaluating ambiguous cases, thereby reducing diagnostic variability. Third, supporting multidisciplinary tumor boards by providing consistent and interpretable data for treatment planning.

The MPS model leverages widely available CT images and H&E-stained slides, ensuring its feasibility for integration into routine clinical workflows. By utilizing ISUP diagnostic tools, the model reduces the need for additional resources or specialized equipment, making it accessible for broad clinical adoption. Its ability to provide reliable predictions of ISUP grading and patient outcomes further underscores its clinical relevance. Beyond its diagnostic capabilities, the MPS model offers significant prognostic value for ccRCC management. It could accurately predict post-surgical OS, enabling clinicians to identify high-risk patients who might benefit from closer monitoring or more aggressive treatment strategies. Unlike many existing prognostic tools, the MPS model complements rather than duplicates clinicopathological factors, ensuring its utility as an additional decision-support tool.

There are some limitations to this study. First, given inherent biases and unknown confounding factors, this retrospective study should be further validated in prospective research. Second, the retrospective extraction of CMP tumor images alone for analysis may have led to the loss of useful information, and future studies should consider integrating features from multiple CT phases. Pathological image diagnosis such as renal biopsy might be more practical. Third, considering the limited availability of CT and digital pathology equipment in some developing and underserved areas, developing more accessible AI models remains an area for future exploration. Fourth, training and developing models for other RCC subtypes, such as papillary RCC, which also use the ISUP grading system, would further refine our RCC nuclear grading diagnostic system.

## Conclusion

This study presents a novel multimodal model that integrates radiomics and pathomics for ISUP grading and prognosis prediction in ccRCC patients. The MPS model demonstrated superior diagnostic accuracy and prognostic value compared to single-modality approaches. With further validation across multiple centers, this MPS system could become a practical tool for clinicians in managing ccRCC patients.

## Data Availability

The datasets generated and analyzed during the current study are available in the TCGA repository, https://portal.gdc.cancer.gov/. The datasets generated and analyzed during the current study are available in the CPTAC repository, https://www.cancerimagingarchive.net/histopathology-imaging-on-tcia/. The RHWU dataset generated and analyzed during the current study is not publicly available due to protect patient privacy.

## Assistance with the study

We thank our colleagues in the Department of Urology and Pathology, Renmin Hospital of Wuhan University, and Baidu Online Network Technology (Beijing) Co., Ltd for their support of this work, as well as all colleagues involved in model development and data collection.

## References

[1] Young M Jackson-Spence F Beltran L. Renal cell carcinoma. Lancet 2024;404:476–91. 39033764 10.1016/S0140-6736(24)00917-6

[2] Jonasch E Walker CL Rathmell WK. Clear cell renal cell carcinoma ontogeny and mechanisms of lethality. Nat Rev Nephrol 2021;17:245–61. 33144689 10.1038/s41581-020-00359-2 PMC8172121

[3] Znaor A Lortet-Tieulent J Laversanne M. International variations and trends in renal cell carcinoma incidence and mortality. Eur Urol 2015;67:519–30. 25449206 10.1016/j.eururo.2014.10.002

[4] Delahunt B Eble JN Egevad L. Grading of renal cell carcinoma. Histopathology 2019;74:4–17. 30565310 10.1111/his.13735

[5] Paner GP Stadler WM Hansel DE. Updates in the eighth edition of the tumor-node-metastasis staging classification for urologic cancers. Eur Urol 2018;73:560–69. 29325693 10.1016/j.eururo.2017.12.018

[6] Moch H Cubilla AL Humphrey PA. The 2016 WHO classification of tumours of the urinary system and male genital organs-part a: renal, penile, and testicular tumours. Eur Urol 2016;70:93–105. 26935559 10.1016/j.eururo.2016.02.029

[7] Dagher J Delahunt B Rioux-Leclercq N. Clear cell renal cell carcinoma: validation of World Health Organization/International Society of Urological Pathology grading. Histopathology 2017;71:918–25. 28718911 10.1111/his.13311

[8] Li S Zhou Z Gao M. Incremental value of automatically segmented perirenal adipose tissue for pathological grading of clear cell renal cell carcinoma: a multicenter cohort study. Int J Surg 2024;110:4221–30. 38573065 10.1097/JS9.0000000000001358 PMC11254242

[9] Yang H Wu K Liu H. An automated surgical decision-making framework for partial or radical nephrectomy based on 3D-CT multi-level anatomical features in renal cell carcinoma. Eur Radiol 2023;33:7532–41. 37289245 10.1007/s00330-023-09812-9 PMC10598088

[10] Browning L Colling R Verrill C. WHO/ISUP grading of clear cell renal cell carcinoma and papillary renal cell carcinoma; validation of grading on the digital pathology platform and perspectives on reproducibility of grade. Diagn Pathol 2021;16:75. 34419085 10.1186/s13000-021-01130-2 PMC8380382

[11] Li S Liao Z He K. Association of sex-specific abdominal adipose tissue with WHO/ISUP grade in clear cell renal cell carcinoma. Insights Imaging 2023;14:194. 37980639 10.1186/s13244-023-01494-7 PMC10657923

[12] Qi YJ Su GH You C. Radiomics in breast cancer: current advances and future directions. Cell Rep Med 2024;5:101719. 39293402 10.1016/j.xcrm.2024.101719 PMC11528234

[13] Wang R Dai W Gong J. Development of a novel combined nomogram model integrating deep learning-pathomics, radiomics and immunoscore to predict postoperative outcome of colorectal cancer lung metastasis patients. J Hematol Oncol 2022;15:11. 35073937 10.1186/s13045-022-01225-3 PMC8785554

[14] Chen S Jiang L Gao F. Machine learning-based pathomics signature could act as a novel prognostic marker for patients with clear cell renal cell carcinoma. Br J Cancer 2022;126:771–77. 34824449 10.1038/s41416-021-01640-2 PMC8888584

[15] Nie P Yang G Wang Y. A CT-based deep learning radiomics nomogram outperforms the existing prognostic models for outcome prediction in clear cell renal cell carcinoma: a multicenter study. Eur Radiol 2023;33:8858–68. 37389608 10.1007/s00330-023-09869-6

[16] Yang G Nie P Yan L. The radiomics-based tumor heterogeneity adds incremental value to the existing prognostic models for predicting outcome in localized clear cell renal cell carcinoma: a multicenter study. Eur J Nucl Med Mol Imaging 2022;49:2949–59. 35344062 10.1007/s00259-022-05773-1

[17] Mahootiha M Qadir HA Bergsland J. Multimodal deep learning for personalized renal cell carcinoma prognosis: integrating CT imaging and clinical data. Comput Methods Programs Biomed 2024;244:107978. 38113804 10.1016/j.cmpb.2023.107978

[18] Bossuyt PM Reitsma JB Bruns DE. STARD 2015: an updated list of essential items for reporting diagnostic accuracy studies. Bmj 2015;351:h5527. 26511519 10.1136/bmj.h5527 PMC4623764

[19] Lu C Xia Y Han J. Multiphase comparative study for WHO/ISUP nuclear grading diagnostic model based on enhanced CT images of clear cell renal cell carcinoma. Sci Rep 2024;14:12043. 38802547 10.1038/s41598-024-60921-x PMC11130204

[20] Vahadane A Peng T Sethi A. Structure-preserving color normalization and sparse stain separation for histological images. IEEE Trans Med Imaging 2016;35:1962–71. 27164577 10.1109/TMI.2016.2529665

[21] Graham S Vu QD Raza SEA. Hover-net: simultaneous segmentation and classification of nuclei in multi-tissue histology images. Med Image Anal 2019;58:101563. 31561183 10.1016/j.media.2019.101563

[22] Delahunt B Cheville JC Martignoni G. The International Society of Urological Pathology (ISUP) grading system for renal cell carcinoma and other prognostic parameters. Am J Surg Pathol 2013;37:1490–504. 24025520 10.1097/PAS.0b013e318299f0fb

[23] Zhu YH Wang X Zhang J. Low enhancement on multiphase contrast-enhanced CT images: an independent predictor of the presence of high tumor grade of clear cell renal cell carcinoma. AJR Am J Roentgenol 2014;203:W295–W300. 25148187 10.2214/AJR.13.12297

[24] Gao J Xu Q Fu Y. Comprehensive evaluation of (68)Ga-PSMA-11 PET/CT parameters for discriminating pathological characteristics in primary clear-cell renal cell carcinoma. Eur J Nucl Med Mol Imaging 2021;48:561–69. 32623502 10.1007/s00259-020-04916-6

[25] Wang G Li L Wang J. Head-to-head comparison of [(68)Ga]Ga-P16-093 and 2-[(18)F]FDG PET/CT in patients with clear cell renal cell carcinoma: a pilot study. Eur J Nucl Med Mol Imaging 2023;50:1499–509. 36600099 10.1007/s00259-022-06101-3

[26] Zheng Q Yang R Xu H. A weakly supervised deep learning model and human-machine fusion for accurate grading of renal cell carcinoma from histopathology slides. Cancers (Basel) 2023;15:3198. 37370808 10.3390/cancers15123198 PMC10296233

[27] Luo Y Liu X Jia Y. Ultrasound contrast-enhanced radiomics model for preoperative prediction of the tumor grade of clear cell renal cell carcinoma: an exploratory study. BMC Med Imaging 2024;24:135. 38844837 10.1186/s12880-024-01317-1 PMC11155131

[28] Shu J Wen D Xi Y. Clear cell renal cell carcinoma: machine learning-based computed tomography radiomics analysis for the prediction of WHO/ISUP grade. Eur J Radiol 2019;121:108738. 31756634 10.1016/j.ejrad.2019.108738

[29] Sun X Liu L Xu K. Prediction of ISUP grading of clear cell renal cell carcinoma using support vector machine model based on CT images. Med (Baltimore) 2019;98:e15022. 10.1097/MD.0000000000015022 PMC6456158 30946334

[30] Li Q Liu YJ Dong D. Multiparametric MRI radiomic model for preoperative predicting WHO/ISUP nuclear grade of clear cell renal cell carcinoma. J Magn Reson Imaging 2020;52:1557–66. 32462799 10.1002/jmri.27182

[31] Yang K Chang S Wang Y. UsingMsfNet to predict the ISUP grade of renal clear cell carcinoma in digital pathology images. Comput Mater Continua 2024;78.

[32] Verine J Colin D Nheb M. Architectural patterns are a relevant morphologic grading system for clear cell renal cell carcinoma prognosis assessment: comparisons with WHO/ISUP grade and integrated staging systems. Am J Surg Pathol 2018;42:423–41. 29356723 10.1097/PAS.0000000000001025

[33] Oza B Eisen T Frangou E. External validation of the 2003 Leibovich prognostic score in patients randomly assigned to SORCE, an International Phase III Trial of Adjuvant Sorafenib in renal cell cancer. J Clin Oncol 2022;40:1772–82. 35213214 10.1200/JCO.21.01090 PMC9148696

